# Selective single-bacteria extraction based on capture and release of microemulsion droplets

**DOI:** 10.1038/s41598-022-19844-8

**Published:** 2022-09-14

**Authors:** Jiyu Li, Dinglong Hu, Chee Kent Lim, Jifeng Ren, Xin Yao, Chao Ma, Marcos, Weiqiang Chen, Patrick K. H. Lee, Raymond H. W. Lam

**Affiliations:** 1 Department of Biomedical Engineering, City University of Hong Kong, Hong Kong, China; 2 Centre for Biosystems, Neuroscience, and Nanotechnology, City University of Hong Kong, Hong Kong, China; 3 Institute of Biointelligence Technology, BGI-Shenzhen, Shenzhen, 518083 China; 4 School of Energy and Environment, City University of Hong Kong, Hong Kong, China; 5 School of Biomedical Engineering, Capital Medical University, Beijing, 100069 China; 6 School of Mechanical and Aerospace Engineering, Nanyang Technological University, Singapore, Singapore; 7 Department of Biomedical Engineering, New York University, New York, NY 10003 USA; 8 Centre for Robotics and Automation, City University of Hong Kong, Hong Kong, China; 9 City University of Hong Kong Shenzhen Research Institute, Shenzhen, 519057 China

**Keywords:** Lab-on-a-chip, Biomedical engineering

## Abstract

Human host-associated microbial communities in body sites can reflect health status based on the population distribution and specific microbial properties in the heterogeneous community. Bacteria identification at the single-cell level provides a reliable biomarker and pathological information for clinical diagnosis. Nevertheless, biosamples obtained from some body sites cannot offer sufficient sample volume and number of target cells as required by most of the existing single-cell isolation methods such as flow cytometry. Herein we report a novel integrated microfluidic system, which consists of a microemulsion module for single-bacteria encapsulation and a sequential microdroplet capture and release module for selectively extracting only the single-bacteria encapsulated in microdroplets. We optimize the system for a success rate of the single-cell extraction to be > 38%. We further verify applicability of the system with prepared cell mixtures (*Methylorubrum extorquens* AM1 and *Methylomicrobium album* BG8) and biosamples collected from human skin, to quantify the population distribution of multiple key species in a heterogeneous microbial community. Results indicate perfect viability of the single-cell extracts and compatibility with downstream analyses such as PCR. Together, this research demonstrates that the reported single-bacteria extraction system can be applied in microbiome and pathology research and clinical diagnosis as a clinical or point-of-care device.

## Introduction

Human host-associated microbial communities in body sites such as gut, skin, mouth, and urogenital track largely determine the disease states of patients^1^. The presence of specific bacterial genera provides not only a reliable biomarker but also pathological information for clinical diagnosis^2^. For examples, primary skin infections, such as impetigo^3^, folliculitis^4^, and boils^5^, are caused by a type of β-hemolytic and coryneform streptococci, known as *Staphylococcus aureus*, under conditions of eczema or insect bites^6^. Another example is skin rash, which is commonly examined via standard skin test, *e.g.* scarping^7^, biopsy^8^, allergy test^9^ and blood test^10^ to determine the skin bacteria compositions in the heterogeneous bacteria community and thus select a narrow-spectrum of antibiotics for the effective treatment. Isolation of the individual responsible organisms in bacterial infection is the key to achieve the corresponding efficient treatment^11^, yet isolating the specific species is technically challenging in terms of manipulation of single bacteria^12^.

Conventional single bacterial cell isolation techniques such as dilution-to-extinction^13^ and single-cell micromanipulation^14^ require extensive labor works. Flow cytometry-based single bacteria isolation^15^ at least a few microliters of the biosample volume, which could be too much to be collected from the infection sites^16^. For instance, flow cytometry on the basis of fluorescence-activated cell sorting (FACS) for single-cell isolation^17^ requires a significant amount of the biosample volume^18^ and indispensable procedures for cell staining which can alter cell properties and limit the more detailed live-cell analyses^19^.

Microfluidics, owing to its capability of precise flow control and micro-particle manipulation, has garnered significant interests on isolation of the single bacterial cells. Specially, microemulsion, which forms stable aqueous microdroplets in an immiscible buffer fluid, usually oil, has been demonstrated as a powerful tool for bacteria isolation and further analyses including incubation, DNA analysis, and drug screening^20–25^. For example, Eun et al*.*^26^ presented the encapsulation of *Escherichia coli* in agarose particles and subsequently analysis by FACS, but the cell extraction for downstream inoculation and analysis was then technically difficult. Marcoux et al*.*^27^ presented an excellent microemulsion strategy to rapidly detect and monitor metabolic activities of single bacteria confined in an emulsified microdroplet. While very effective, the fully packed microdroplet array in the downstream of the microfluidic device requires very precise controls of the physical and chemical properties to maintain the droplet stability; and extraction of the single-bacteria encapsulating microdroplets without further staining or processes (*e.g.*, enzymatic reactions) is difficult. In addition, Boedicker et al*.*^28^ presented a microemulsion system for rapid detection and drug susceptibility screening of bacteria in complex biological matrices. However, the generated droplets in these methods cannot be extracted and demulsified individually for the more traditional single bacteria analyses, such as polymerase chain reaction (PCR) amplification and DNA sequencing. Moreover, Liu et al*.*^29^ developed a microfluidic emulsion-based device for incubation of single bacteria isolated from a mixture of *P. curdlanolyticus* and *E. coli.* However, the operation configuration has not been optimized for the higher isolation rate of single cell encapsulation and a microdroplet elimination scheme for the void and multi-bacteria droplets is required. Therefore, an integrated microfluidic system that achieves efficient isolation of bacteria at a single-cell level from a limited input of biosample and is highly compatible with various downstream analyses via controllable cell extraction is urgently needed yet largely unavailable.

Recently, we have developed a microfluidic strategy for sequential capture of mammalian cells (10–20 μm) in aqueous biosamples, *e.g.* blood, in microfluidic based platform^30,31^ with a capture rate of > 95%. Cells are sequentially captured in the micro-sieve structures, whose positions have been arranged carefully according to the deterministic lateral shift^32^ for the maximum capture rate. Notably, on top of the additional functional capability offered by microemulsion, the cell-encapsulating microdroplets enlarge the physical scale (from sub-micron for bacteria to tens of micron for microdroplets) of the ‘particles’ to be manipulated. Hence, we hypothesized that integration of a microemulsion unit for encapsulation of bacteria in a droplet of which the working scale is like our previous device will extend its application for single-bacteria manipulation and subsequent genetic and functional analyses.

In this work, we developed a micro-device integrated with microemulsion and sequential micro-sieves for single-bacteria encapsulation and selective cell extraction. We optimized the system dimensions and operation parameters by characterizing the microdroplet generation, the sequential microdroplet trapping, the single-bacteria encapsulation in droplets and the microdroplet release. As a proof-of-concept, we demonstrate applicability of the micro-device with prepared skin bacteria and human skin biosample and achieved single-bacteria extraction from multi-species bacterial communities and the following basic genetic analysis. We believe our integrated microfluidic system will demonstrate a useful and powerful tool for clinical diagnosis^33^ of microbial infection and as well for screening potential drugs or antibiotics.

## Results and discussion

### Device operation

**Figure 1:**
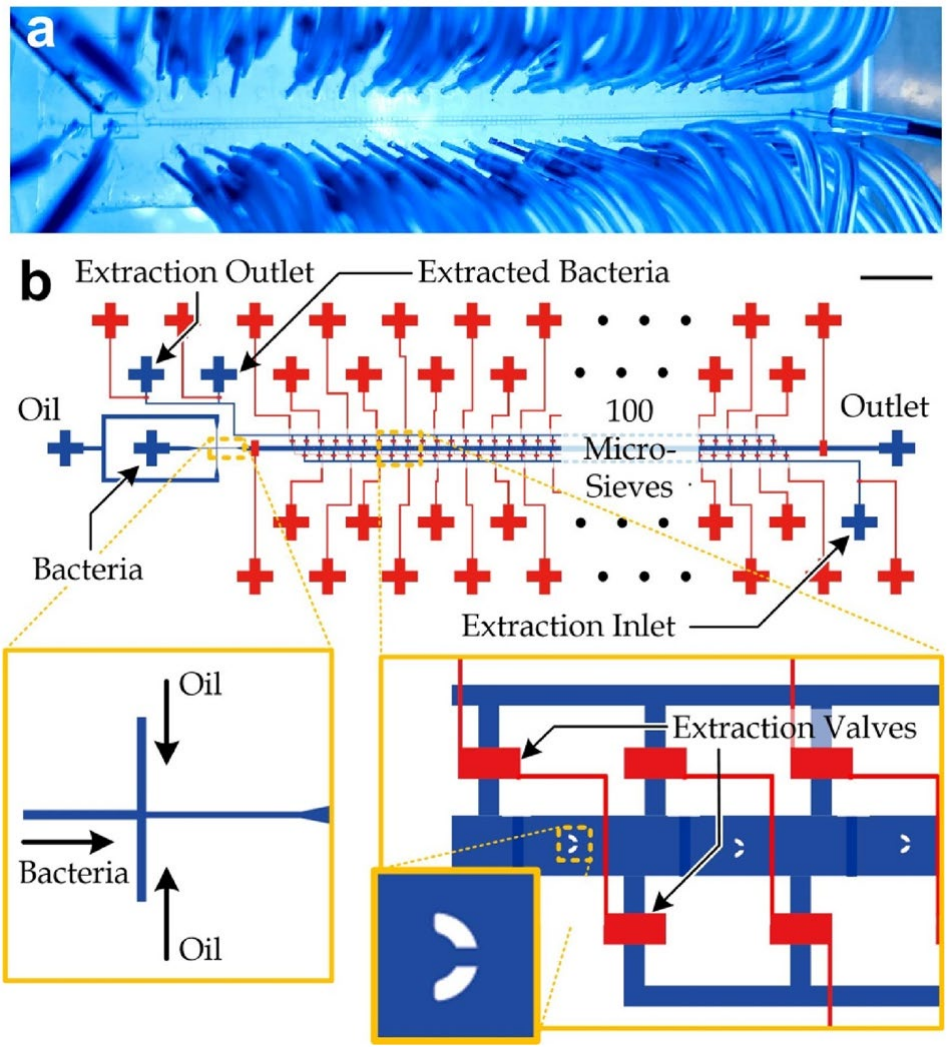
(**a**) A fabricated single-bacteria extraction micro-device. (**b**) Device design. Scale bar: 2 mm. The left inset shows the microchannel junction for microdroplet generation via microemulsion. The right inset is the microchannel section containing a series of micro-sieves and multiple pairs of microvalve-gated side channels for selectively extracting the target microdroplets.

We have designed and fabricated (as described in Methods) a microfluidic device for encapsulation and extraction of single bacteria (Fig. 1a,b). The device consists of a microemulsion module for generating cell-encapsulating microdroplets, a series of micro-sieves for microdroplet capture, and multiple pairs of side microchannels for droplet release. In the microemulsion module, a bacteria sample was inserted from the central inlet and buffer mineral oil was applied along the side channels (Fig. 1b, *left* inset). We applied only the culture medium along the central biosample microchannel and mineral oil (M8410, Sigma) mixed with 5% (volume ratio) surfactant (Span 80, S6760, Sigma) along the side buffer oil microchannels (nozzle width: 10 μm). The surfactant was added to stabilize and reduce the interfacial tension between the oil and generated droplets (~ 2 mN/m)^34,35^.

The biosamples were handled carefully in the preparation and operation steps to minimize unnecessary loss. Before the device operation, we utilized a syringe filled with the biosample and connected it to the ‘Bacteria’ inlet via silicon tubing; followed by slowly injecting the biosample until it reached near the cross junction of the microemulsion module. We then used a metal binder clip to clamp the tubing to prevent the biosample flow. Afterward, the buffer mineral oil was injected via the ‘Oil’ inlet to fill up all the rest microchannels of the device. Inlet pressures of the biosample and the buffer oil should be set to the desired pressure right before device operation. Once the metal paper clip was removed, the biosample could flow to the cross junction and emulsify as microdroplets. At the beginning, there were a few microdroplets significant bigger or smaller than the average droplet size, yet the microdroplet size became stable after a sample flow of < 4 nl.

The droplets then entered the microchannel region (width: 145 μm; height: 30 μm) and were captured in the micro-sieves (Fig. 1b, *right* inset). We have adopted our previously reported micro-sieve placement scheme^31^ for capturing the microdroplets. The working principle is based on placing each micro-sieve at a position such that a flowing particle has a proper range of the lateral distance with centers of micro-sieves. The particle can flow into and be captured in an empty micro-sieve but flow around an occupied micro-sieve on one pre-defined lateral side. The micro-sieve arrangement can offer a high capture rate (~ 95%), though the sequence of the trapped droplets is not the major concern. We considered a working droplet diameter of 20.35 ± SD 1.3 µm and performed the simulation study (Fig. S1) to determine the alternating lateral displacements (5 um to the *left/right* side) and dimensions (outer diameter: 45 μm; inner diameter: 25 μm; opening gap width: 10 μm) of the micro-sieves. Besides, the channel height (30 µm) and working flow rates (~ 0.25 μl/min) of the buffer mineral oil (kinematic viscosity: 20.5 mm^2^/s) implies a low *Reynolds* number (≪ 1) flow.

We supplied a sample volume of < 20 nl (including the loss of < 4 nl in the preparation step as mentioned above) for generating and capturing ~ 87.7 ± SD 3.15 microdroplets (Fig. S2). The microdroplets containing only one stained bacterial cell can be identified under a fluorescence microscope. The corresponding pair of side micro-valves (Fig. 1b, *right* inset) were then ungated in sequence along the extraction microchannels and apply a flow from the extraction inlet with a total volume of 2 µl to export one microdroplet to a PCR tube placed at the extraction outlet.

The microfluidic bacteria encapsulation and extraction device supports multiple rounds of operation, for achieving higher isolation rates and reproducibility. Before the next round of operation, the sample flow can be gated with a metal binder clip at the corresponding inlet tubing. The remaining captured microdroplets, which may contain no or multiple cells, should all be released to the sample outlet for cleaning up all the micro-sieves. The micro-valve between the microemulsion module and the micro-sieve channel is ungated and the micro-valves for the side extraction channels are gated to restore the device condition for the next round of operation.

### Flow conditions for micro-droplet generation

**Figure 2:**
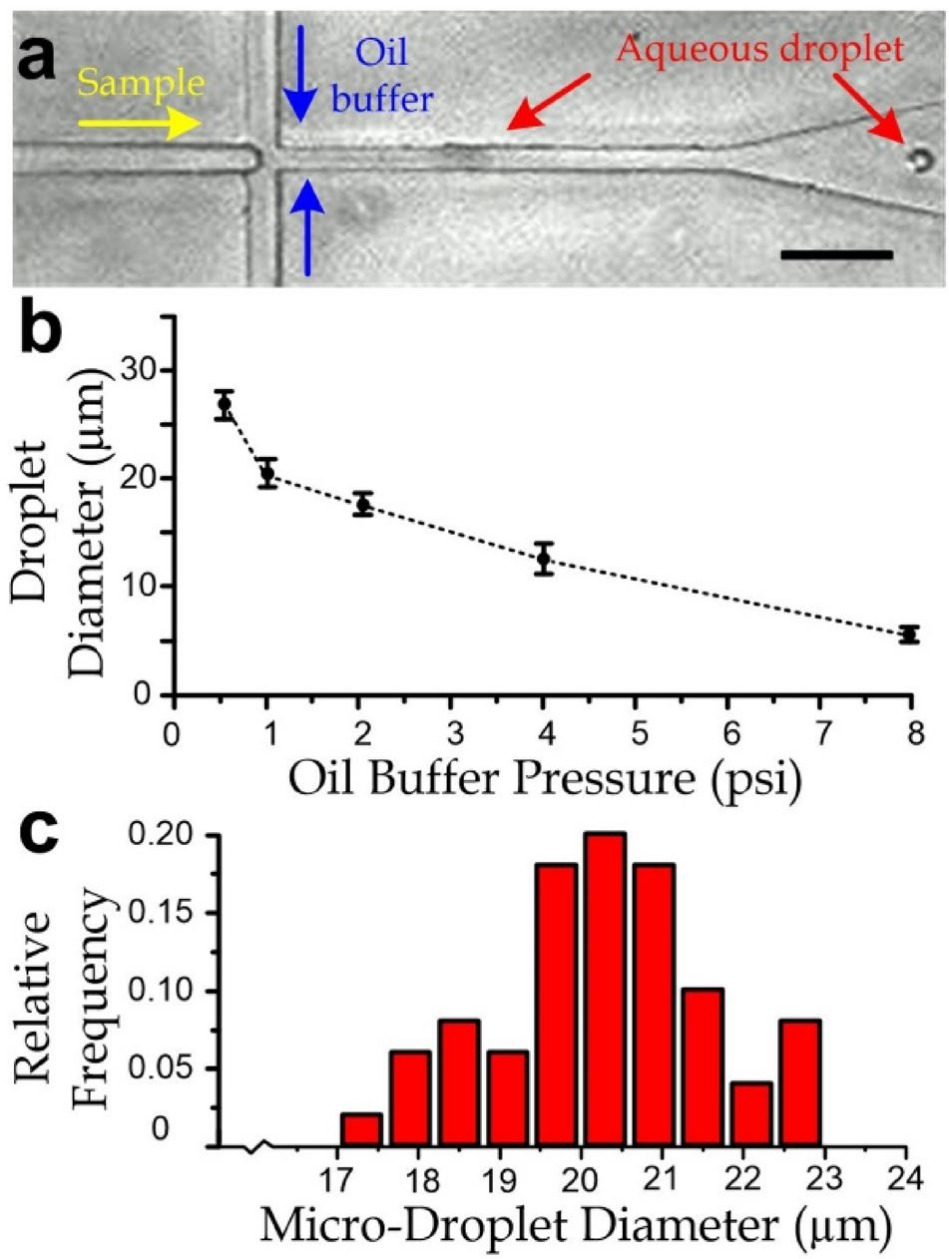
(**a**) Micrograph of microfluidic emulsion. Scale bar: 100 µm. (**b**) Diameter of the generated microdroplets for different gage pressures of oil and a consistent biosample pressure of 0.5 psi. Error bars are the standard deviations. (**c**) Statistics of diameter of the generated micro-droplet under a 1 psi driving pressure of the sample flow.

We conducted experiments to characterize diameters of the generated micro-droplets as a function of the buffer oil pressure (0.5–8 psi), whereas the biosample pressure was maintained 0.5 psi. Diameters of the generated droplets under the different pressure ratios were quantified from video snapshots (Fig. 2a) captured by a camera (Zyla 5.5, Andor) under a microscope (TE300, Nikon). Our results suggest that a higher driving pressure induced droplets with smaller diameters (Fig. 2b) at a higher generation rate (Fig. S3). Each constant pressure level of the oil buffer generated droplets with a range of diameters. For example, the buffer oil pressure of 1 psi can generate droplets at a rate of 2.13 ± SD 0.46 droplets/s, with a diameter of 20.35 ± SD 1.3 µm, also indicating a low polydispersity index (defined as standard deviation over diameter) of 0.0639 as exhibited in Fig. 2b,c.

Additionally, because the microemulsion process involves the balance between the interfacial tension stress and the liquid shear stress, the corresponding critical Weber number (*We*) should be maintained in the order of one. We can consider *We* = *τR*/(2*σ*), where *R* is the droplet radius, *σ* (~ 2 mN/m for water and oil with 5% Span 80^35^) is the interfacial tension, and *τ* is the shear stress in the continuous phase liquid (oil). *τ* should be proportional to the oil viscosity (~ 10 mm^2^/s^36^), and the driving pressures. Hence, a larger *τ* caused by a higher driving pressure should induce a smaller *R*, agreeing with our results (Fig. 2b).

### Flow configuration for droplet capture

We investigated the droplet deformation under different pressure levels across a micro-sieve region by both experiments and simulation analyses. The simulation model depicted a representative droplet-capture microchannel section (length: 400 μm). It contained one micro-sieve with the lateral distance of 200 µm from the channel section center. Simulated results for the cases of an ‘empty’ micro-sieve and (Fig. S1a) a micro-sieve capturing a microdroplet (Fig. S1b) suggest that a separating distance of 400 µm between micro-sieves is sufficiently long for flow recovery that the deviation of flow direction could restore to a negligible amount (< 5%).

**Figure 3:**
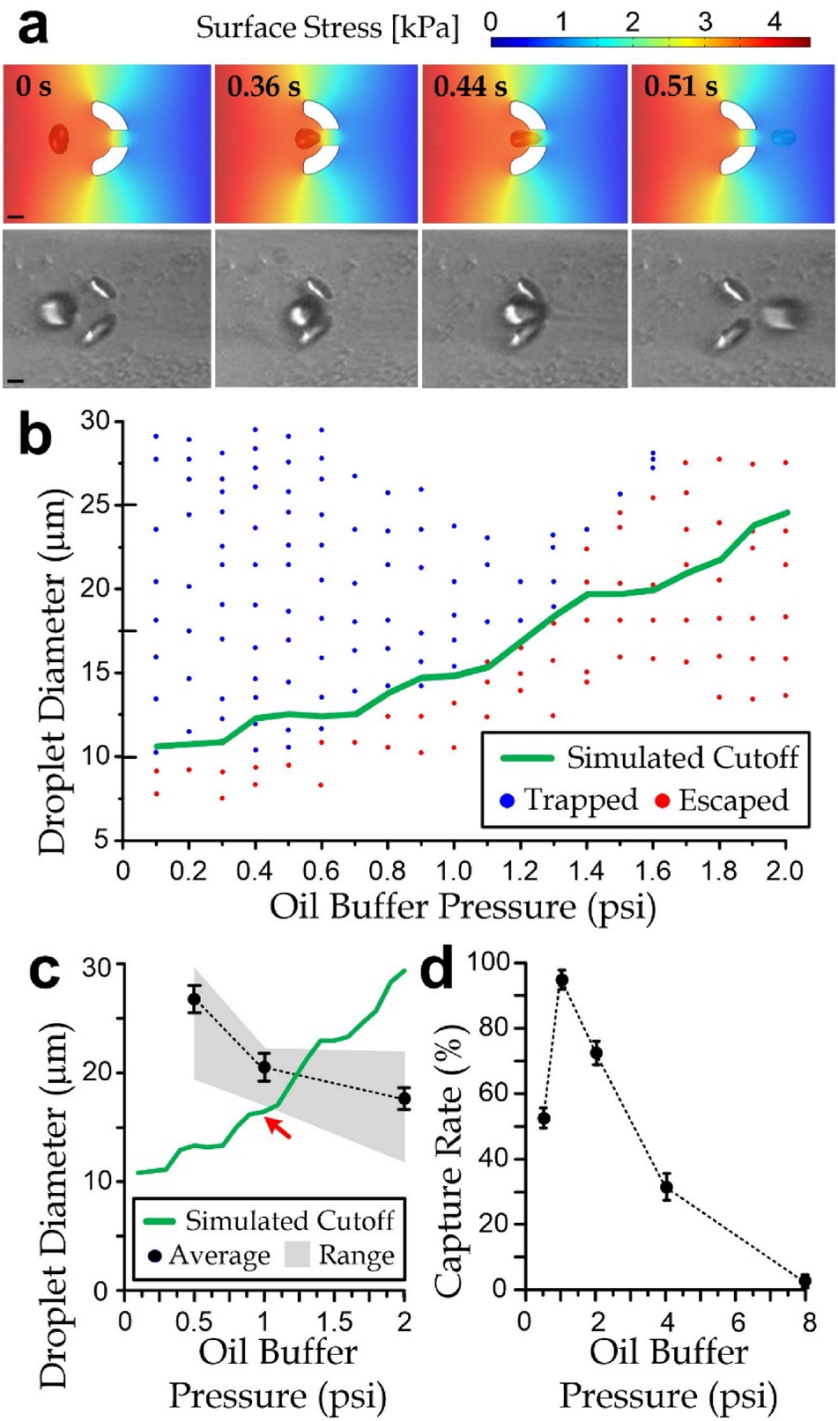
(**a**) Cell shape and position at different reference time points obtained by simulation (upper) and experiments (lower), driven by an exceeding pressure of 2 psi such that the cell could deform and squeeze through the gap of the micro-sieve. Scale bar: 5 µm. (**b**) Counts of trapped and escaped droplets upon different diameters and oil buffer pressures. The green line specifies the simulated cutoff droplet diameter for different oil pressures, determining whether the droplet can be trapped (above the cutoff line. (**c**) Averages (points) and ranges (gray region) of generated droplet diameter for different oil pressures. (**d**) Capture rate of micro-droplets under different oil pressures.

To predict the maximum working buffer pressure level for successful encapsulation of a droplet with a defined diameter, we also conducted simulation by including the deformation and movement of a trapped droplet for different diameters. As illustrated in Fig. 3a (upper row), a droplet larger than the micro-sieve gap can still deform and pass through the gap under an excessive pressure level. The simulation results estimate the maximum pressure levels for trapping different droplet diameters as shown in Fig. 3b.

Further, we performed experiments to verify the simulation results. We applied the microemulsion at different buffer pressure levels to generate droplets and examine whether the droplet can be trapped or escape via the micro-sieve gap as shown in Fig. 3a (lower row). The diameter of generated droplets can be varied by tuning the sample inlet pressure in the range of 0.3–0.6 psi. We then recorded the capability of droplet trapping for different droplet diameters and buffer pressure levels as plotted in Fig. 3b. The simulation and experimental results match reasonably for the buffer pressure ≤ 1.3 psi as the simulated cutoff could roughly separate the trapped and escaped droplet cases. Yet, for the higher buffer pressure, the simulation is no longer representative, possibly because the larger deformation of microdroplets under the higher pressure can no longer be considered as solid as adopted in our simulation.

To achieve the maximum droplet capture rate, we selected the buffer pressure level to be able to trap for the entire diameter range of the generated droplets. The capture rate (*R*_*c*_) is considered as the percentage of the number of captured droplets (*N*_*c*_) compared to the total number of droplets (*N*) flowing in the channel, *i.e.*, *R*_*c*_ = 100% × *N*_*c*_/*N*. Recalling Fig. 2c that the droplet diameter is > 17 µm for 1 psi buffer pressure, we adopted 1 psi for driving the oil buffer flow, as indicated in Fig. 3c (arrow). We have also quantified for the capture rate of the generated droplets under different buffer pressures as shown in Fig. 3d, indicating that the ideal buffer pressure level for the maximum capture rate (~ 95%) is indeed around 1 psi. As predicted, the capture rate reduces for the pressure higher than 1 psi because the droplets can escape from the micro-sieve gaps. A lower capture rate for the pressure below 1 psi could be explained by the exceeded lateral shift of the generated oversized droplets.

**Figure 4:**
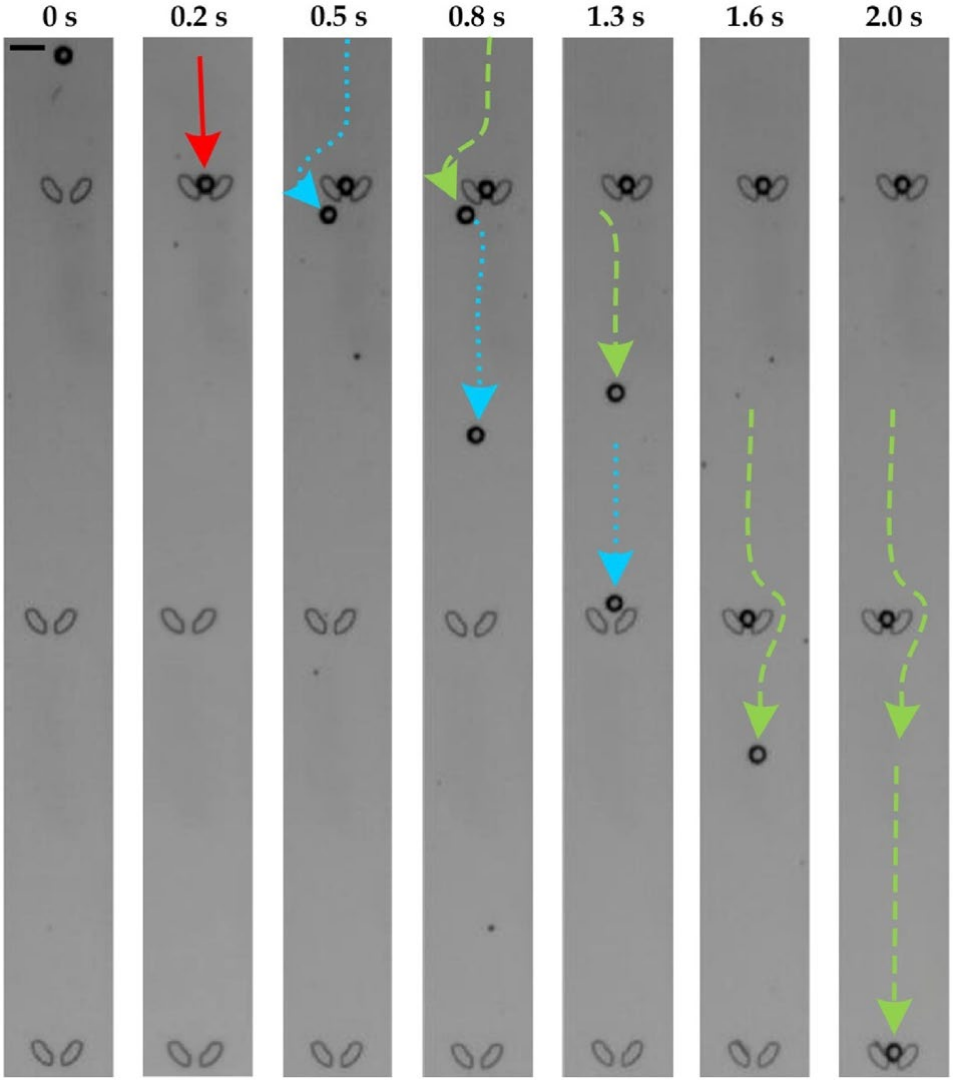
Trajectories of three droplets being captured sequentially in micro-sieves. Scale bar (upper left): 30 μm.

Furthermore, we have verified that the configuration of microstructure dimensions and operation parameters can maintain sequential trapping of the generated microdroplets in micro-sieves as shown in Fig. 4 and Supplemental Video 1. We have also examined that the single droplets can maintain in the micro-sieves stably, *i.e.*, without separated into and combined with multiple droplets. As mentioned previously, each micro-sieve is placed at a position such that there is a lateral distance between its center and a coming droplet. Hence, a flow droplet can either enter an empty micro-sieve or flow around an occupied micro-sieve, of which the micro-sieve wall can prevent physical contacts between the flowing droplet and the captured droplet. In other words, the device design and operation can ensure that the microdroplets are always separated without combining multiple of them together. (We have never seen any combination of multiple droplets in the device so far.) On the other hand, we have also analyzed that the droplet can maintain as one body given the viscosity and interfacial tension effects dominating over the inertial effects. Here, we adopt a widely used relation^37^ on the critical diameter for the droplet breakdown, based on the comparison between scales of the viscosity-dominated effect and the inertia-dominated effect. This implies that the droplet diameter (*D*) needs to be sufficiently small for eliminating the droplet break-down, *i.e.*, *D* < 2*μ*^2^/(*σρ*), where *σ* (~ 2 mN/m^35^) is the interfacial tension, *μ* (~ 10 mm^2^/s^36^) is the oil viscosity and *ρ* (0.85 g/cm^3,38^) is the oil density. In fact, the critical diameter (= 2*μ*^2^/(*σρ*) = 118 μm) is much larger than the diameter of the generated microdroplets (20.35 ± SD 1.3 µm) in this work. Overall, our analysis supports that a captured droplet can maintain stably in a micro-sieve of the device.

### Single-bacteria-encapsulating microdroplet capture and release

**Figure 5:**
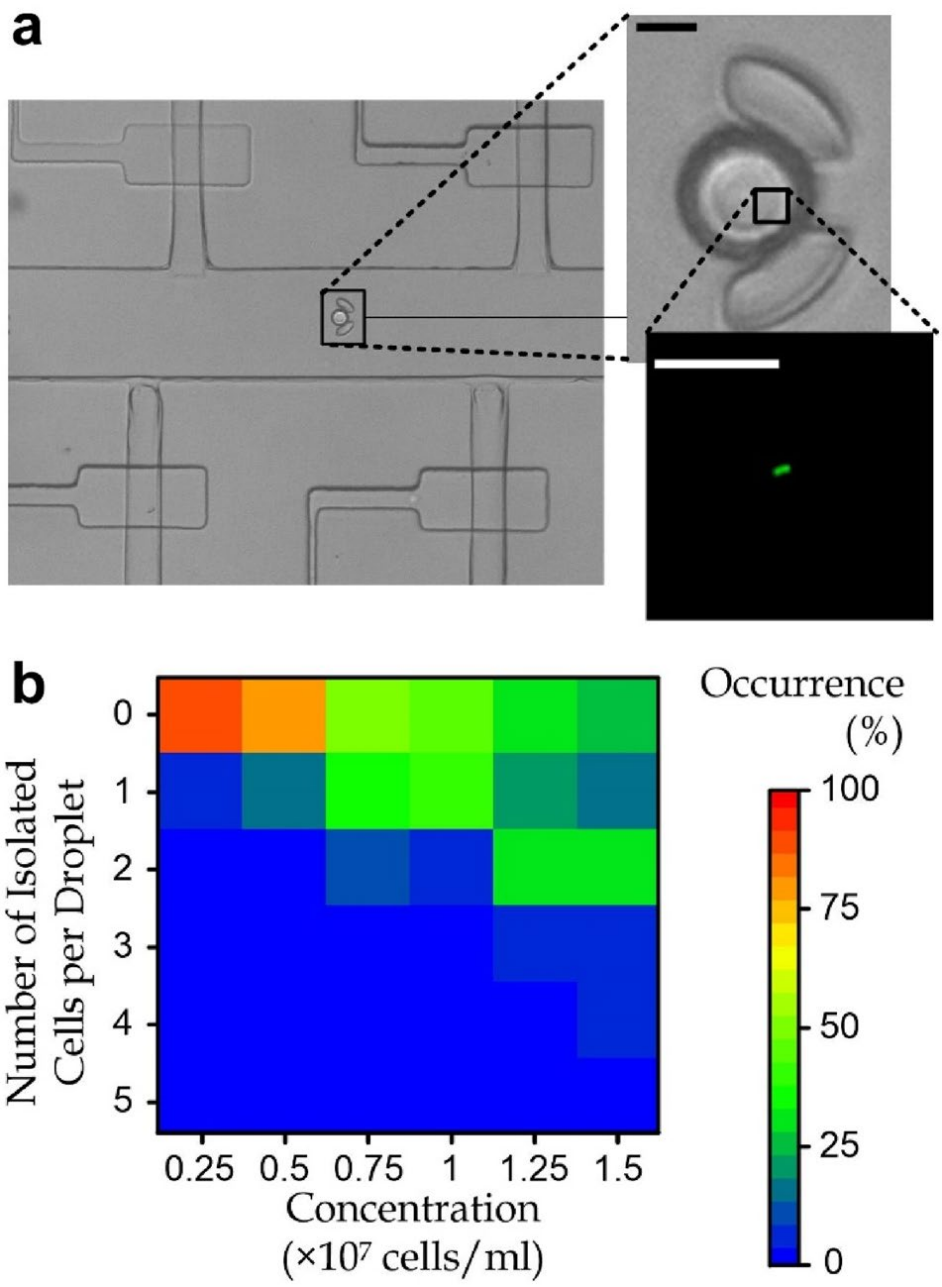
(**a**) A micro-sieve contains a micro-droplet encapsulating a rod-shaped *M. extorquens* AM1 cell, labeled with SYTO™ 9. Scale bar: 10 μm. (**b**) Occurrence of different numbers of isolated bacteria in a microdroplet upon cell concentration in the bio-sample.

We performed experiments to characterize the rate of microdroplet captured in the micro-sieves with different cell concentrations in biosamples. For each experiment run, a sample volume of 11.3 ± SD 0.594 nl on top of the < 4 nl loss in the preparation step, could generate ~ 95.75 ± SD 0.96 droplets. We counted the generated micro-droplets during the microemulsion process. Afterward we counted the microdroplets captured in the micro-sieves as shown in Fig. 5a. The capture rate of the microdroplet is 94.54 ± SD 0.322%, demonstrating a high capture efficiency of our design of the micro-sieve array.

We further tested different cell concentrations (0.25 × 10^7^–1.5 × 10^7^ cell/ml) of pre-stained *Methylorubrum extorquens* AM1 (Fig. 5b and Fig. S4) to identify the range for a higher possibility of generating the single-cell-encapsulated micro-droplets. Here, we define the droplet-isolation rate (*R*_*dr*_) as the percentage of the number of single bacterial cells each being encapsulated in a microdroplet (*n*_*se*_) compared to the total number of bacterial cells appearing in all the captured microdroplets (*n*), *i.e.*, *R*_*dr*_ = 100% × *n*_*se*_/*n*. We found that the highest droplet-isolation rate is 42.86% with the cell concentration of 1 × 10^7^ cell/ml. Considering the random arrangement of bacteria in the biosample, the probability of the number of encapsulated bacteria in a generated microdroplet should be roughly described by the Poisson distribution and the maximum chance of having a single cell captured in a droplet is ~ 37%^39^. For instance, Hirama and Torii have reported the generation and pairing of two single-cell-encapsulating droplets inside a bigger secondary droplet with a success rate of 20%^40^, implying that the chance of generating a single-cell-encapsulating droplet is ~ 45%, with a good agreement with the reported droplet-isolation rate in this work. On the other hand, it should be noted that such droplet-isolation rate does not consider those droplets escaped from the device; and hence a device-isolation rate (*R*_*de*_), defined by the number of single bacteria each in a captured droplet compared to the number of cells entered the device, can be estimated by the product of the capture rate and the droplet-isolation rate, *i.e.*, *R*_*de*_ = *R*_*c*_*R*_*dr*_ (/100%). Considering also the ~ 95% success rate of the microdroplet capture, a device-isolation rate of > 40% was obtained, *i.e.*, > 40% of bacteria in the biosample can appear as a single-cell in a micro-sieve-captured microdroplet.

**Figure 6:**
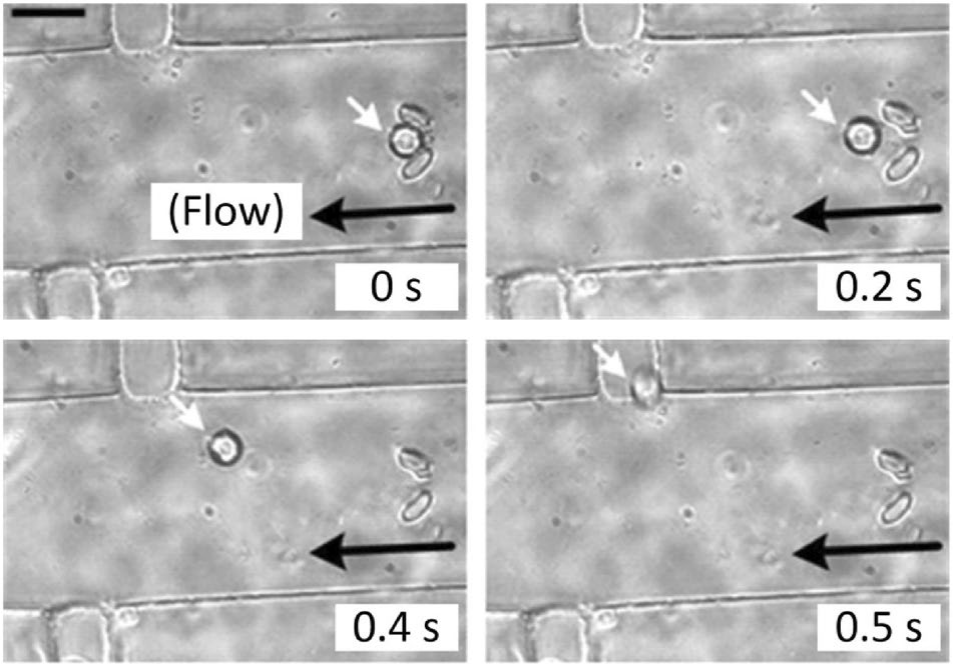
Extraction of a target microdroplet (white arrow) containing single bacterial cell at different time points. Scale bar: 50 µm. Flow from the extraction inlet induces a backward flow along the micro-sieve region (black arrow) such that the microdroplet escape the micro-sieve and escape the device through the side channel outlet.

Beside capturing droplets containing single bacteria, extraction of target droplets encapsulating single-bacteria for further analysis such as clinical diagnosis is also critical^34^. As demonstrated in Fig. 1, the micro-sieve is positioned between its corresponding pair of inlet and outlet channels with the lateral sieve location is further set along. As expected, the stimulation streamlines of the side-channel flow can easily release the droplet from the micro-sieve accordingly with an input flow (Fig. S5). In this case, the captured droplet can be individually extracted by opening the micro-valves of the side channels with the device inlet and outlet closed, followed by applying flow through the side channels with a total volume of 2 µl under a driving pressure of 0.5 psi (Fig. 6 and Supplemental Video 2), to transfer the cell-containing droplet to a syringe tube placed at the corresponding extraction outlet. Then, a culture medium (volume: 500 μl) was added in the syringe tube was vortex-shaken and centrifuged, followed by pipetting away the upper layer of mineral oil. Each isolated cell can then be transferred to another growth environment for further incubation or analysis.

**Figure 7:**
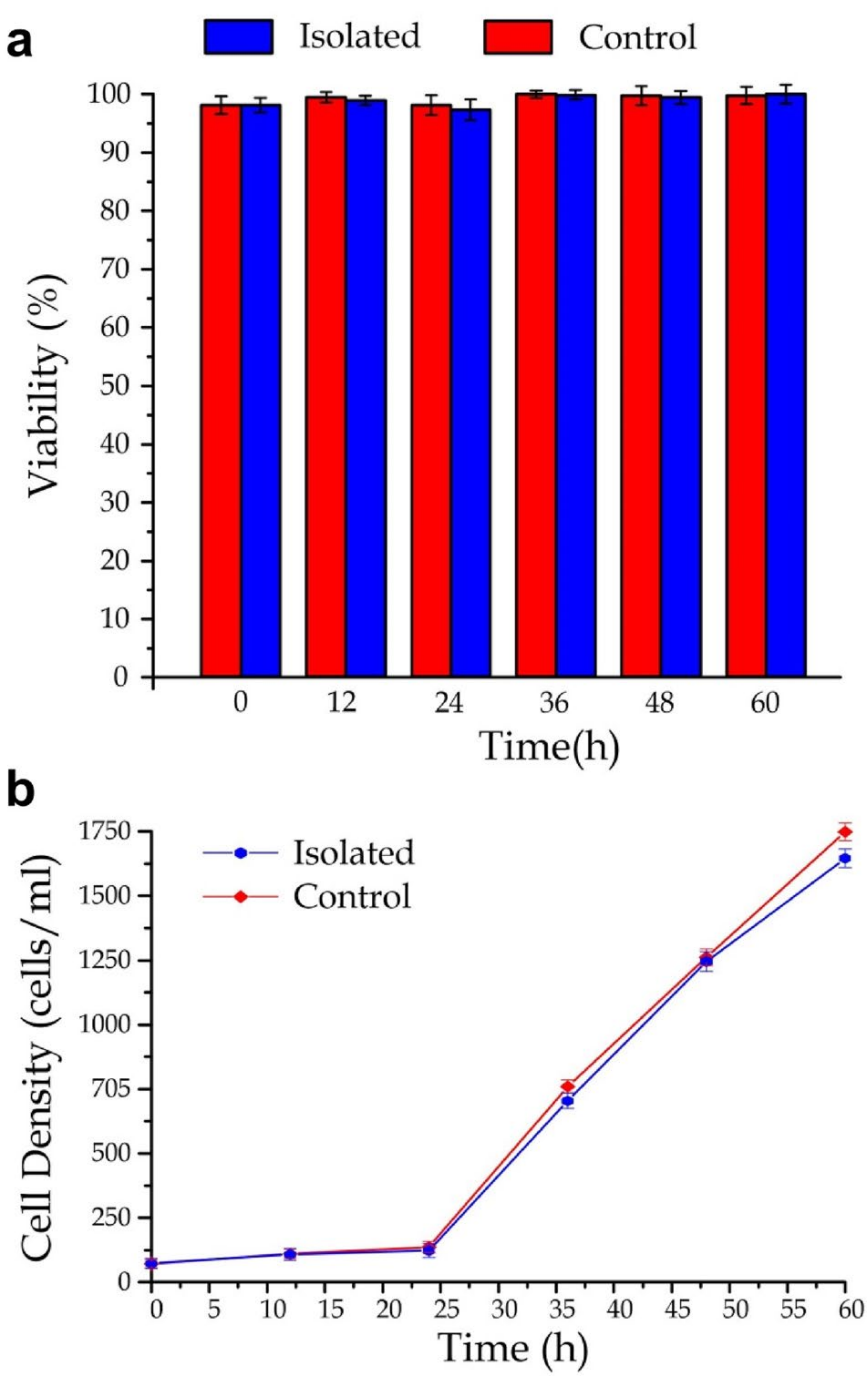
(**a**) Viability and (**b**) density of *M. extorquens* AM1 bacteria (with and without processed by the single-bacteria isolation and extraction) over different culture periods from 12 to 60 h (*N* = 4 for each point).

We further investigated viability of individual cells in the released droplets. *M. extorquens* AM1 cells were collected from the extraction outlets after the cell isolation process. It should be mentioned that the entire cell encapsulation and extraction process should be within 1 h to minimize unexpectedly high toxicity of the surfactant. Each isolated single bacterium was collected with an individual syringe tube placed at the extraction outlet. Each syringe tube was then amended with the antibiotic kanamycin (50 mg/ml) and methanol (v/v 1.3%) as the carbon source. The syringe tubes were then placed in in a shaking incubator (30 °C and 200 rpm). After a culture period of 12 h to 60 h, the bacteria culture was stained (LIVE/DEAD BacLight Bacterial Viability Kit, Thermo fisher, USA) and quantified for cell density and viability by the flow cytometry (FASCVerse, BD, USA). For comparison, we also applied the regular bacteria culture, in which the desired singe cells were prepared by simple dilution, as the control group. Our results (Fig. 7) indicate that the reported cell isolation technique can perfectly maintain the cell viability and growth as the regular culture. Viability of the extracted single bacteria (*R*_*v*_), defined as the percentage of the number of live cells compared to the total cell number, is > 95% during the culture period (Fig. 7a). Here, we may consider the baseline of the chance of bacteria being able to be extracted individually and grow to be the overall ‘isolation rate’ (*R*), which can be calculated as the product of the device-isolation rate and the measured cell viability, *i.e.*, *R* = *R*_*de*_*R*_*v*_ (/100%). This isolation rate can be considered as a value for comparison with other previously reported single-bacteria isolation techniques; and the single-bacteria isolation technique presented here can achieve an isolation rate of > 38%, comparable to other widely used but laborious techniques^16,41^).

### Identification of single cells from multi-bacterial strain mixtures

As a proof-of-concept, we applied the device to extract single-bacteria from known multi-strain biosamples and unknown multi-strain biosamples and verified its bacteria identification by PCR tests. First, we prepared a known-strain sample by mixing *M. extorquens* AM1 (prelabeled with DAPI) and *M. album* BG8 (prelabeled with propidium iodide) with a 1:1 population ratio at a resultant cell density of 1 × 10^7^ cell/ml. In this case, the color of extracted bacteria observed in microscope can reflect which species they belong to and be considered as the confirmation of the PCR results obtained later. In addition, we performed another experiment to generate microdroplets of culture media without cells as the negative control. The PCR results (Table 1 and Fig. S7) indicate that 67% of the isolated *M. extorquens* single cells and 88% of the isolated *M. album* single cells could be amplified and correctly identified. There was no misidentification of the type of bacteria and there were no signals for the control case, *i.e.*, no false positive result. However, not all the sorted single cells could yield a positive PCR amplification from the isolated bacteria. This could be caused by factors that cells in the extracted droplets may fail to be lysed and genomic amplification with only one bacterium is a challenging task without a guaranteed success rate^42^.

**Table 1:** PCR results of the sorted single cells from a defined two-species mixture.

PCR primers	Isolated sample	
	*M. extorquens* AM1 single cells	*M. album* BG8 single cells
*M. extorquens* AM1-specific	16/24^a^ (67%)	0/24 (0%)
*M. album* BG8-specific	0/24 (0%)	21/24 (88%)

^a^The number of positive PCR amplification over the number of single cells tested.

To further validate the applicability for analyse of clinical biosample, we then adopted the same single-bacteria extraction procedures on human skin biosamples (collection protocol detailed in Methods) to conducted PCR for the gene sequences of the isolated single-cells. We have performed five repeated rounds of the cell extraction and identification experiments on different days from the same subject. The average number of single bacteria extracted from a successful round is ~ 41 cells. The droplet capture rate was 97.3 ± SE 0.21% and the single-bacteria droplet-isolation rate was 44%, agreeing with the values for other experiments described above. We performed PCR targeting the 16S ribosomal RNA gene on the sorted single cells; and 23% of them yielded amplification products which were subsequently sequenced, whereas those remaining unsuccessful cases were possibly due to inadequate DNA content of single bacteria for the PCR process.

Among the successfully sequenced single bacteria, the gene sequence analysis revealed that 67% of the isolated skin bacteria belonged to the bacterial genus *Cutibacterium* (> 95% similarity) (GenBank Accession no.: OM250408; OM250409; OM250410; OM266150), which is known to be a human skin commensal^43^. The other 17% of bacteria were the *Variovorax guangxiensis*^44^ (> 99% similarity) (GenBank Accession no.: OM250407), which is present in the common environment. The remaining 16% of the collected bacteria were other unknown strains. These results essentially demonstrates compatibility of the bacteria isolation technique with PCR, and probably with other downstream genetic analyses^45^, such as rRNA sequencing^46^ and metagenomics^47,48^. Under the condition that the bacteria are much smaller than the droplet size, in principle presence of the bacteria does not affect mechanics of the microemulsion process. Once the bio-sample is diluted in an aqueous medium with a working range of cell density, the cell isolation strategy is applicable for other microbial species with different sizes and shapes with the isolation rate maintained. Furthermore, this single-bacteria isolation technique can also be applied to other human liquid biopsies as a minimally invasive method for disease diagnoses. For examples, meningococcal infection can be identified by examining bacteria in peripheral blood^49^ instead of directly obtaining the cerebrospinal fluid by surgery. Osteogenesis-associated microbial contamination in bone^50^ can be predicted by obtaining the population distribution of different periapical periodontitis-related strains in saliva for the increased number of Epstein–Barr virus and some anaerobic bacteria (e.g. *Porphyromonas gingivalis*, *Aggregatibacter actinomycetemcomitans*, *Tannerella forsythia*, or *Prevotella intermedia*)^51^.

For the further system development, the throughput of the reported single-cell encapsulation and extraction method can be improved. For examples, the device design can be further extended as a longer microchannel containing more micro-sieves. Computer-assisted image analysis algorithm can be developed to recognize the number of encapsulated bacteria in a droplet captured in a micro-sieve, monitored under a microscope. The microfluidic operations can be further automated for faster pneumatic microvalve switching and movements of the movable microscope stage for recognition of single cell encapsulating droplets. On the other hand, the limited isolation rate (< 45%) correlated with the Poisson distribution can be improved by multiple strategies, such as the pre-ordering of bacteria cells before microemulsion^52^ for an isolation rate of > 80%. Alternatively, we can recollect the residual volume biosample from the device outlet for another round of single bacteria isolation process, implying that multiple rounds of the single-bacteria isolation process by recycling the biosample can achieve a higher single cell isolation rate.

## Materials and methods

### Fabrication

The single-bacteria extraction micro-device is fabricated by multilayer soft lithography^53^, as summarized in Fig. S9. Layers of AZ 50XT (AZ-50XT, AZ Electronic Materials) or/and SU-8 (SU-8 2010, Microchem) photoresists on 4-inch silicon wafers are micropatterned photolithography to generate replica molds. In particular, the ‘control layer’ and mold contains SU-8 (height: 10 µm) microstructures on a wafer. To fabricate the ‘flow layer’ mold, SU-8 (height: 30 µm) is micropatterned on a wafer, followed by micropatterning AZ 50XT (height: 25 µm) overhead. The reflow process (120 °C, 1 min) is then applied to refine cross-sections of the AZ 50XT microstructures, for the complete closure of microvalves in the resultant micro-devices fabricated as described below. These two replica molds are treated by air plasma (energy: 5 kJ; PDC-002, Harrick Plasma) and deposited with trichloro (1H, 1H, 2H, 2H-perfluoro-octyl) silane (Sigma-Aldrich) for better release of the molded materials.

Polydimethylsiloxane (PDMS) monomer and the curing agent (Sylgard-184, Dow Corning) are mixed (10% weight ratio), degassed. The PDMS pre-polymer is poured onto the flow layer mold with a thickness of 3 mm and spin-coated onto the control layer mold with a thickness of 40 µm. The PDMS pre-polymer samples are then cured at 80 ºC for 2 h. The molded PDMS substrates are then cut and peeled off. Air plasma treatment (energy: 10 kJ) is applied to the PDMS surfaces, followed by aligning and bonding them under a dissection microscope. The bonded PDMS is cut, peeled off, punched at the inlets/outlets, treated with air plasma (energy: 10 kJ) and bonded on a glass slide.

### Flow simulation

Computational studies are performed using COMSOL Multiphysics (COMSOL, Burlington, MA). Two three-dimensional models are constructed and analyzed for the flow characteristics with and without presence of a micro-droplet (diameter: 20 μm) captured in a micro-sieve. They are both a channel section containing a serious of micro-sieves positioned along the channel with an alternating lateral offset along the consecutive sieves. The inlet pressure and outlet pressure of both models are defined as 0.2758 kPa and 0 kPa, respectively.

On the other hand, a three-dimensional model of a microchannel section containing a micro-sieve and a moving and deforming droplet is applied to determine the critical droplet diameter for the effective droplet capture. We consider the laminar two-phase flow for the droplet (water) and its surrounding liquid (mineral oil). The gage inlet pressure applied to the microchannel section should be ~ 1% of the inlet pressure of the device because each device contains a hundred of the micro-sieve sections consecutively. To reveal the dynamic droplet deformation during the encapsulation process, we adopt the moving mesh module for the time-lapsed computation. As the droplet may undergo a large deformation, we configure the hyper-elastic mesh smoothing for the droplet body.

### Stained bacteria mixture

*Methylorubrum extorquens* AM1 (ATCC 14718)^43^ and *Methylomicrobium album* BG8 (ATCC 33003)^54^ were purchased from the American Type Culture Collection. They were cultivated aerobically in 160-ml serum bottles in the modified *Methylococcus* medium (ATCC medium 1057), in which CuSO_4_·5H_2_O was replaced by CuCl_2_·2H_2_O, MnSO_4_·H_2_O was replaced by MnCl_2_·4H_2_O, FeSO_4_ was replaced by FeSO_4_·7H_2_O, and 0.5% (v/v) methanol was added. The bottles were loosely capped and incubated at 30 ℃ with shaking (200 rpm). Stationary phase cells were collected by centrifugation (9600×*g*). 70% isopropanol was used to disrupt cell membranes for staining. *M. album* BG8 were stained with 0.3 μM 4′,6-diamidino-2-phenylindole (DAPI; Thermo Fisher Scientific, USA) and *M. extorquens* AM1 with 60 μM propidium iodide (Invitrogen, USA). The cell concentration was measured by a hemocytometer (Isolab, Germany). The two bacteria were mixed with an equal concentration (1 × 10^7^ cell/ml) prior to experiments.

### Bacteria extraction from human skin

Skin samples were collected from the forearm of a 28-year-old healthy Chinese male. As human subject is involved, the bacteria extraction procedures and the required ethical matters were approved by the Committee on the *Use and Care of Animals* of the City University of Hong Kong (study: A-0236). A written informed consent was also obtained from the volunteer. The collection of skin samples was approved by the City University of Hong Kong Human Subjects Ethics Sub-Committee (ref: H001553). Briefly, a sterile swab (Isohelix, UK) moistened in phosphate-buffered saline (Life Technologies, UK) was used to sample a 4-cm^2^ area on the forearm in a back-and-forth motion for 40 times. Cells dislodged from a swab by vortex mixing. The cells were then stained by 20 µM SYTO™ 9 (Thermo Fisher Scientific, USA). The collected sample were then filtered by a syringe filter (0.2 μm, Sigma) to remove the skin cells. The cell concentration was measured by a hemocytometer (Isolab, Germany) and adjusted to 1 × 10^7^ cells/ml before experiments. We confirm that all methods were performed in accordance with the relevant guidelines and regulations. In the bacteria isolation, totally 500 droplets were extracted for PCR amplification (90%) then sequenced.

### Polymerase chain reaction (PCR)

The isolated cells are trapped in microdroplets held within mineral oil with a volume of 2 μl, which were thermally lysed and mixed with PCR reagents. We identified the known species *M. extorquens* AM1 with the primer pair q_phaB-Mex^55^, and *M. album* BG8 with the primer pair QpmoA-7^56^ using Premix Ex Taq Ver. 2.0 (Takara, Japan) PCR reagent. On the other hand, for unknown species in the human skin extracts, we applied the universal 16S ribosomal RNA gene primer set 63F and M1387R^57^ with Phusion High-Fidelity PCR Master Mix with HF Buffer (New England Biolabs, USA). The 16S ribosomal RNA gene amplicon analysis using Sanger sequencing with the 63F primer was conducted on the positive PCR products excised and purified from the agarose gel after electrophoresis. We then searched the resultant nucleotide sequences with the NCBI nr database via BLASTn, to identify the corresponding stains.

### Statistics

Error bars in plots represent standard deviations if not additionally specified. Two-tailed *p*-values are calculated by Student’s t-test. An asterisk shown in plots represents a significant difference (*p* < 0.05) between two data groups.

## Conclusion

We successfully developed a single-bacteria extraction system based on microemulsion and deterministic lateral displacement of the generated micro-droplets sequentially flowing into micro-sieves. We demonstrated the single-cell isolation from heterogeneous microbial communities using our developed system can then be achieved with a small biosample volume of ~ 10 nl. We have also demonstrated the isolation of single bacteria from a prepared bacteria mixture of *M. extorquens* AM1 and *M. album* BG8 and verified the unaffected viability. This demonstrates the compatible of our integrated microfluidic system with conventional downstream bioanalysis processes such as incubation, genetic detection, and biochemical analysis. More importantly, our microfluidic single-bacteria extraction system was able to quantify the key bacteria population of human skin samples, highlighting its potential applications in other clinical and environmental heterogenous microbial samples. In summary, we envision that our microfluidic single-bacteria isolation system, with further system automation, will demonstrate a self-functioning medical device and achieve clinical and point-of-care diagnostic applications.

## Supplementary Information

Supplementary Information 1.

Supplementary Video 1.

Supplementary Video 2.

## Data Availability

The DNA sequences adopted in this work have been submitted to INSDC member repository. The GenBank accession numbers are OM250407, OM250408, OM250409, and OM250410 for *Cutibacterium*, and OM266150 for *V. guangxiensis*.
