# Supplementary material for: Selective single-bacteria extraction based on capture and release of microemulsion droplets
Source: Sci Rep. 2022 Sep 14;12:15461. doi: 10.1038/s41598-022-19844-8 (PMC9474873; doi:10.1038/s41598-022-19844-8)
Supplement: Supplementary file 1 — Supplementary Information 1. [file 41598_2022_19844_MOESM1_ESM.docx]

**Supplementary Figures**

Selective Single-Bacteria Extraction Based on Capture and Release of Microemulsion Droplets

Jiyu Li^1,2,#^, Dinglong Hu^1,3,#^, Chee Kent Lim^4^, Jifeng Ren^1,5^, Xin Yao^6^, Chao Ma^7^, Marcos^6^, Weiqiang Chen^7^, Patrick K. H. Lee*^,2,4^ and Raymond H. W. Lam*^,1,2,8,9^

^1^ Department of Biomedical Engineering, City University of Hong Kong, Hong Kong, China

^2^ Centre for Biosystems, Neuroscience, and Nanotechnology, City University of Hong Kong, China

^3^ Institute of Biointelligence Technology, BGI-Shenzhen, Shenzhen 518083, China

^4^ School of Energy and Environment, City University of Hong Kong, Hong Kong, China

^5^ School of Biomedical Engineering, Capital Medical University, Beijing 100069, China

^6^ School of Mechanical and Aerospace Engineering, Nanyang Technological University, Singapore

^7^ Department of Biomedical Engineering, New York University, New York, NY 10003

^8^ Centre for Robotics and Automation, City University of Hong Kong, Hong Kong, China

^9^ City University of Hong Kong Shenzhen Research Institute, Shenzhen 519057, China

*Correspondence should be addressed to R.H.W. Lam (email address: rhwlam@cityu.edu.hk; Tel: +852-3442-8577; Fax: +852-3442-0172) and P.K.H. Lee (email address: patrick.kh.lee@cityu.edu.hk; Tel: +852-3442-4625; Fax: +852-3442-0688)


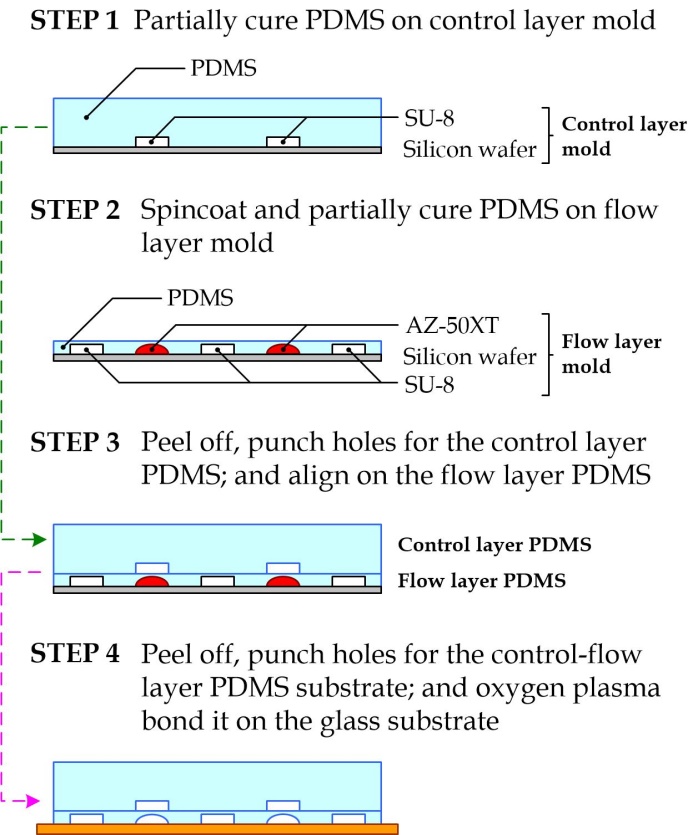


**Figure S1.** Fabrication process for the microfluidic single-bacteria isolation device.


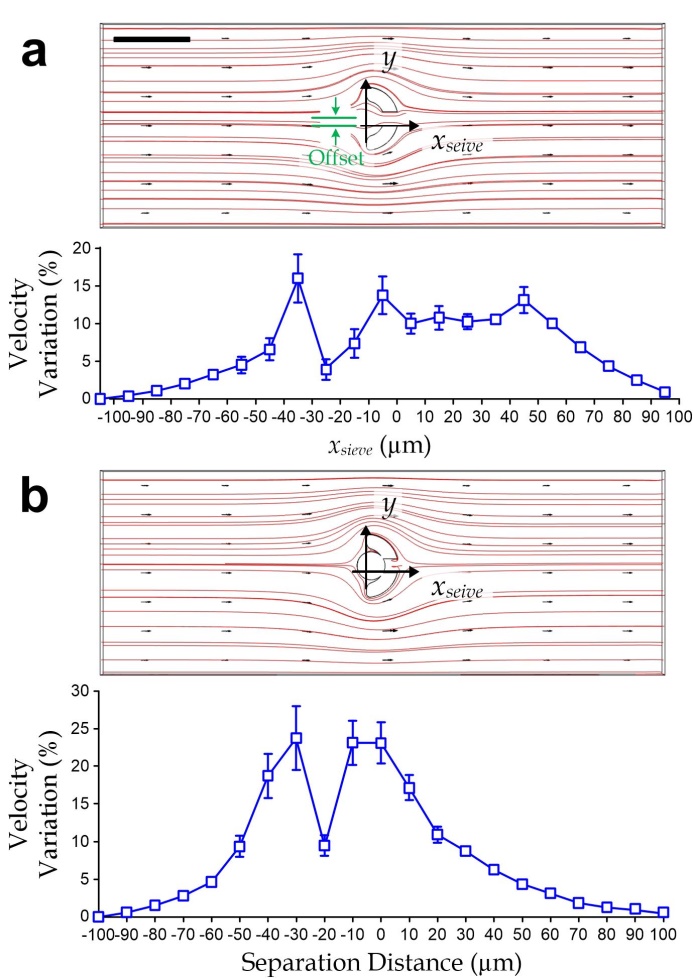


**Figure S2.** Simulated velocity profiles and velocity variation profiles for (**a**) an unoccupied micro-sieve and (**b**) an occupied micro-sieve. The velocity profiles include local velocities (arrow) and streamlines (*red* line) in the mid-height top view cross-section of an isolation microchannel section. Scale bar: 50 μm. The velocity variations along the isolation microchannel section are also plotted against a separation distance from the *x*-position of micro-sieve. We further quantified the velocity profile as a dimensionless quantity as the average value of the relative velocity magnitude deviating from the flow along the microchannel length, average($\sqrt{(v_{y}^{2}+v_{z}^{2})/(v_{x}^{2}+v_{y}^{2}+v_{z}^{2})}$), where *v_x_*, *v_y_* and *v_z_* represent the velocity components *x*-axis (channel length), *y*-axis (channel width) and *z*-axis (channel width), respectively, at a location inside the microchannel region.


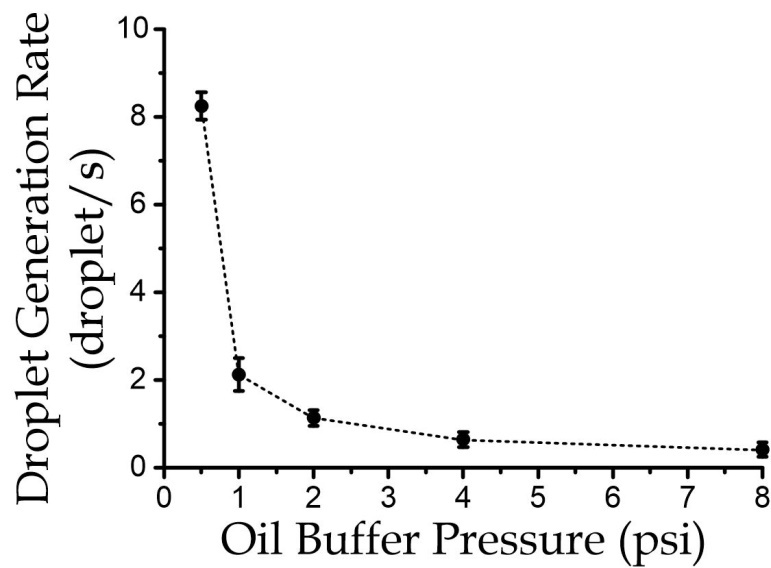


**Figure S3.** Generation rate of micro-droplets for different levels of the oil buffer inlet pressure (*N* = 4).


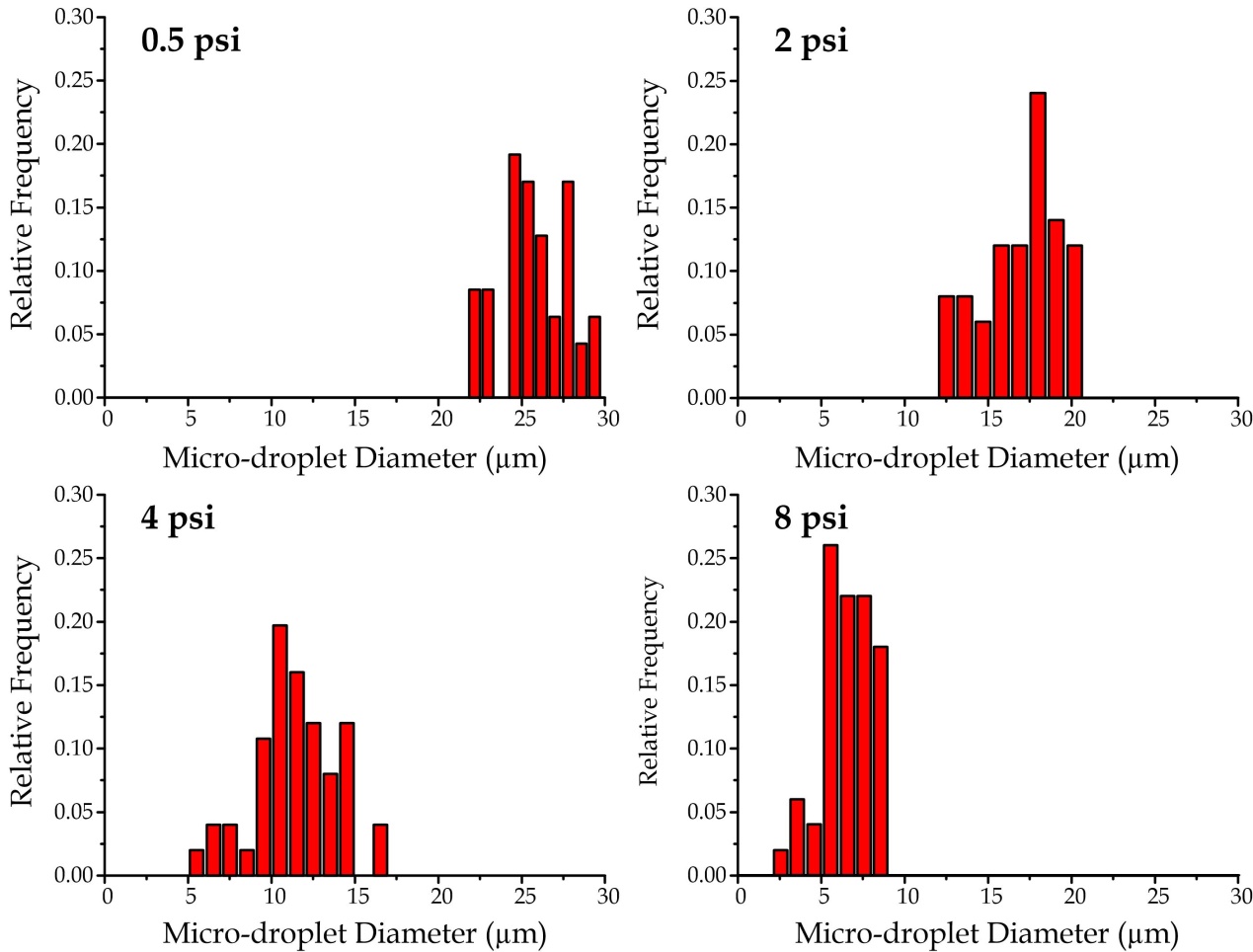


**Figure S4.** Diameter distributions of micro-droplet generated under the oil buffer pressure level of 0.5 psi, 2 psi, 4 psi and 8 psi.


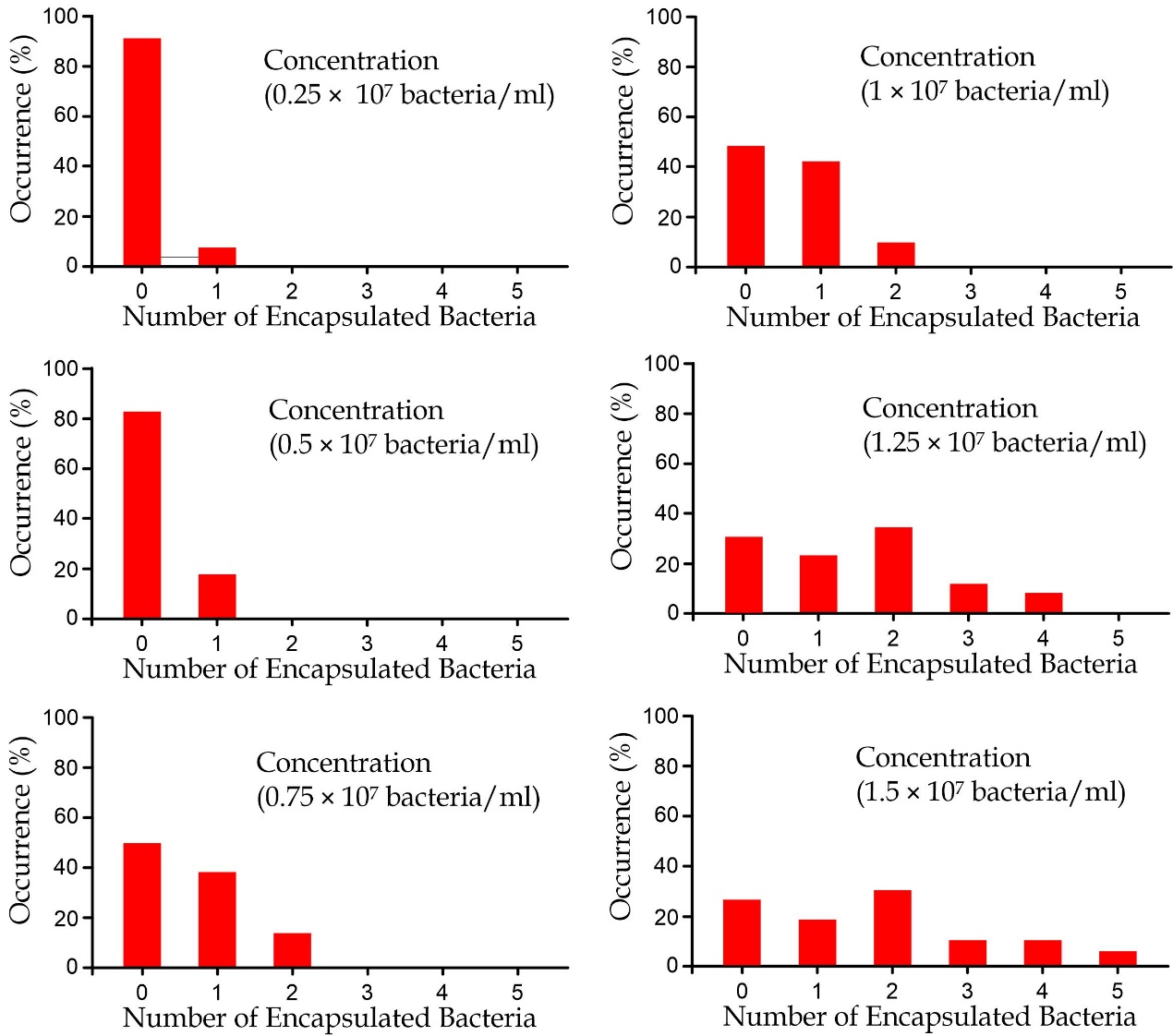


**Figure S5.** Statistics of the number of encapsulated bacteria in a generated micro-droplet upon different bacteria concentrations in the biosample.

**
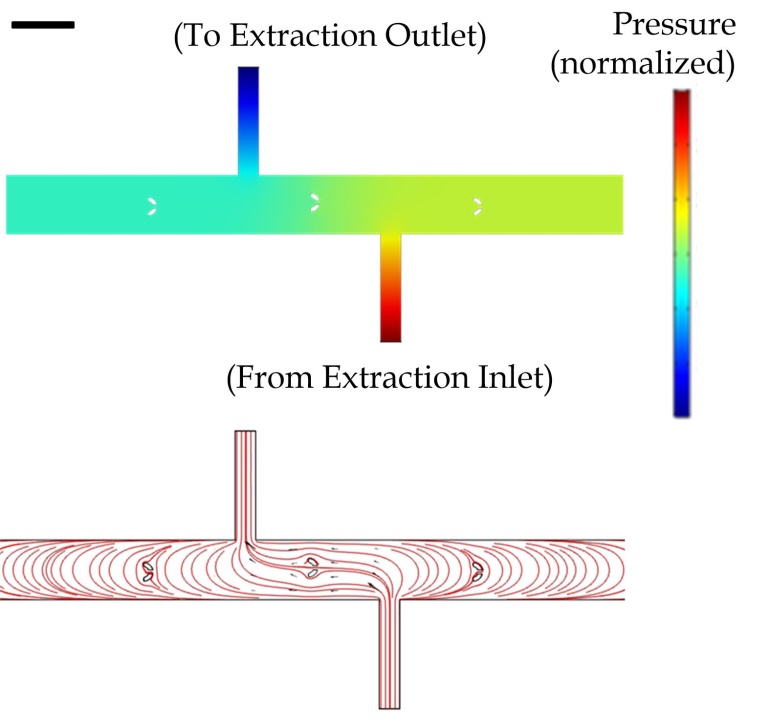
**

**Figure S6.** Simulated pressure profiles (*upper*) and streamlines (*lower*) of mineral oil flowing from the extraction inlet along a microchannel section containing one micro-sieve. The two adjacent micro-sieves (on the left and on the right) are included for matching the device structure. Scale bar: 100 μm.

**a**


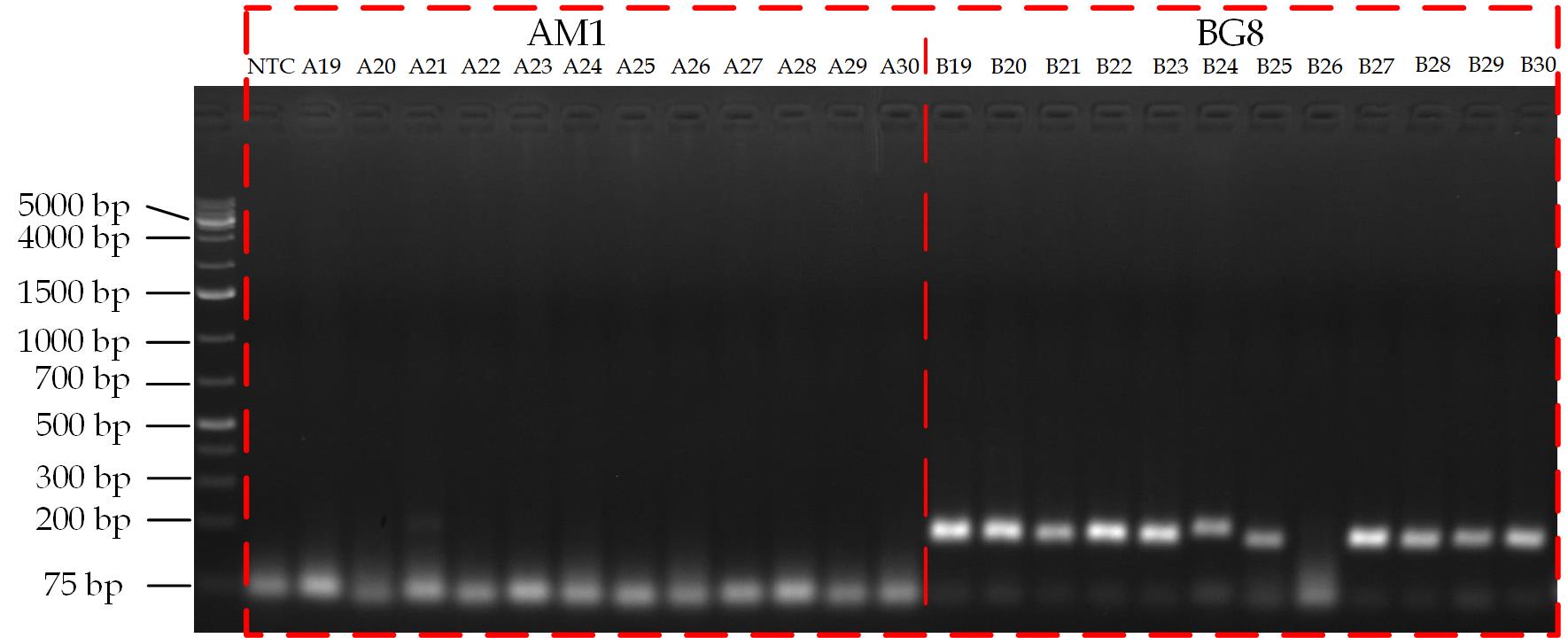


**b**


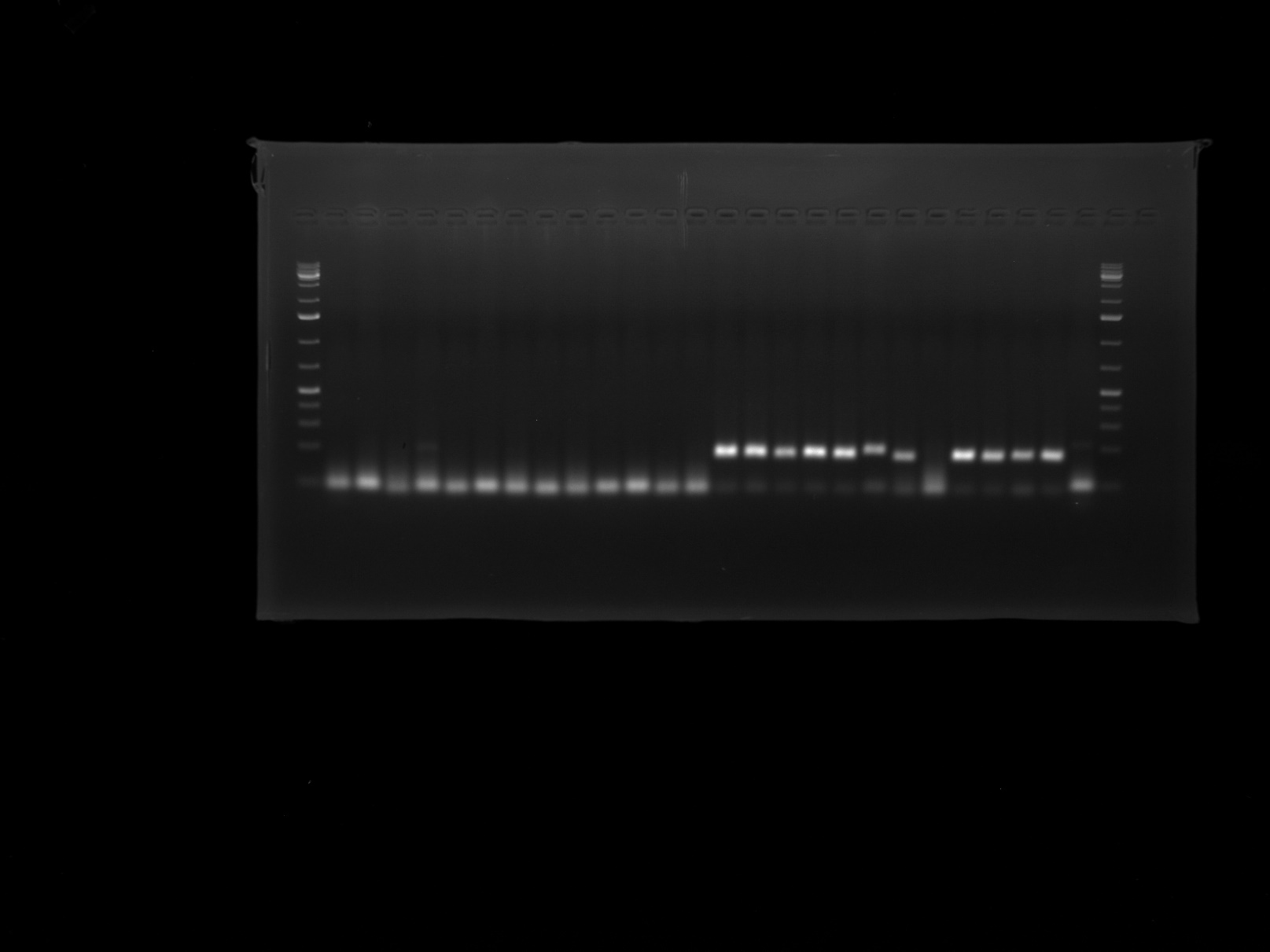


**Figure S7.** (**a**) PCR band profiles of isolated individual cells of *M. extorquens* AM1 (11 cells, labeled as A19 to A30) and *M. album* BG8 (11 cells, labeled as B19 to B30) using the QpmoA-FWD-7 and QpmoA-REV-7 primer set specific for *M. album* BG8. NTC corresponds to non-template control. (**b**) Original gel image, captured by the Azure C600 imaging system (Azure Biosystems, Dublin, California, United States) at an ultraviolet wavelength of 302 nm.
